# Pre-post changes in psychosocial functioning among relatives of patients with depressive disorders after Brief Multifamily Psychoeducation: A pilot study

**DOI:** 10.1186/1471-244X-11-56

**Published:** 2011-04-11

**Authors:** Fujika Katsuki, Hiroshi Takeuchi, Mizuho Konishi, Megumi Sasaki, Yuka Murase, Atsuko Naito, Hiroko Toyoda, Masako Suzuki, Nao Shiraishi, Yosuke Kubota, Yoshiko Yoshimatsu, Toshiaki A Furukawa

**Affiliations:** 1 Department of Psychiatric and Mental Health Nursing, Nagoya City University School of Nursing, Nagoya, Japan; 2 Department of Psychiatry and Cognitive-Behavioral Medicine, Nagoya City University Graduate School of Medical Sciences, Nagoya, Japan; 3 Department of Psychology, Tokaigakuin University, Gifu, Japan; 4 Center for Education and Research on the Science of Preventive Education Naruto University of Education, Tokushima, Japan; 5 Department of Nursing, Nagoya City University Hospital, Nagoya, Japan; 6 Shiseikai Yagoto Hospital, Nagoya, Japan; 7 Tosei General Hospital, Department of Neuropsychiatry, Aichi, Japan; 8 Kyoto University Graduate School of Medicine/School of Public Health, Department of Health Promotion and Human Behavior, Kyoto, Japan

## Abstract

**Background:**

Depressive disorder is often chronic and recurrent, and results in a heavy psychosocial burden on the families of patients with this disorder. This study aims to examine the effectiveness of brief multifamily psychoeducation designed to alleviate their psychosocial burden.

**Methods:**

Thirty-two relatives of patients with major depressive disorder participated in an open study testing the effectiveness of brief multifamily psychoeducation. The intervention consisted of four sessions over the course of 6 weeks. Outcome measures focused on emotional distress, care burden and Expressed Emotion (EE).

**Results:**

The emotional distress, care burden and EE of the family all showed statistically significant improvements from baseline to after the family intervention. The proportion of relatives scoring 9 or more on K6, which indicates possible depressive or anxiety disorder, decreased from sixteen relatives (50.0%) at baseline, to only 3 relatives (9.3%) after the intervention.

**Conclusions:**

This study suggests that brief multifamily psychoeducation is a useful intervention to reduce the psychosocial burden of the relatives of patients with depressive disorder. Further evaluation of family psychoeducation for relatives of patients with depressive disorder is warranted.

## Background

Major depressive disorder (MDD) is a long-lasting illness with significant effects on the patient's family, social, and work life [1,2]. Treatment failure results in a low recovery rate and frequent relapses [3]. According to studies on the naturalistic course of MDD, a prospective study in Japan showed that 10-20% of patients entering treatment remain chronically depressed without recovery up to 1 or even 2 years [4]. Once recovered, the cumulative probability of remaining well without subthreshold symptoms was 57% at 1 year, 47% at 2 years and 35% at 5 years [5]. Additionally, individuals with MDD have a higher level of divorce [6] and severe financial strain [7].

It is easy to imagine that relatives of these patients with MDD are fraught with heavy psychosocial burden and show increased rates of depression and anxiety [8,9]. Research suggests that approximately 40% of caregivers of patients with chronic psychiatric disorder have mental disturbance [10,11]. Among relatives of patients with MDD, the patient's behavior and mood disturbance and relative's emotional distress were associated with the relatives' burden [12]. Fadden et al. [13] reported that the burden of relatives of patients with MDD included restrictions in social and leisure activities, a fall in family income, and a considerable strain on the marital relationship. Relatives of patients with depression found some of the behavior of the patients to be difficult to bear, and the relatives had negative consequences such as misery, withdrawal, and worrying which commonly caused problems; however, few relatives know how to deal with the difficult behavior of patients [13].

Family psychoeducation is recognized as part of the optimal treatment for patients with psychotic disorder [14,15]. This intervention has been shown to reduce the rates of relapse and hospitalization among individuals with psychotic disorders and is recognized as an evidenced-based treatment for psychotic disorder [16,17]. Several randomized controlled trials have found that family psychoeducation is effective in enhancing the course of bipolar disorder [18-21] and MDD [22-24]. In adolescent major depression, patients in the group who experienced family psychoeducation showed greater improvements in social functioning and adolescent-parent relationship than the control group [24]. Among patients with recurrent MDD during long-term antidepressant treatment, patients who were treated with the family intervention approach based on the McMaster Model had a significantly lower relapse rate than patients who were treated with dose increase and clinical management [22].

In this open study, we aimed to investigate if and how brief multifamily psychoeducation alleviated the family psychosocial burden among the relatives of patients with major depression.

## Methods

### Participants

The participants of this study met the following criteria. The participants were: (i) relatives of persons who met the criteria for MDD according to *The Diagnostic and Statistical Manual *(DSM-IV) based on the consensus rating by the psychiatrists in charge, (ii) relatives of persons who were inpatients or outpatients of the Department of Psychiatry at Nagoya City University Hospital, and (iii) relatives who provided written informed consent. The relatives of patients with MDD who had a concurrent diagnosis of primary anxiety or personality disorder, substance abuse or dependence were excluded. Relatives of patients who met the DSM-IV criteria for bipolar disorder were allowed to participate in the intervention groups but were excluded from the present study.

### Treatment

The family intervention program, which we called 'brief multifamily psychoeducation', is based on the McFarlane Model [25], the Evidence-Based Practices Toolkit for Family Psycho-Education [26], and the standard model of the Japanese Network of Psychoeducation and Family Support Program [27]. The brief multifamily psychoeducational program consisted of four sessions. Each of the four multifamily psychoeducational program groups consisted of the relatives of approximately six patients. The staff consisted of one or two psychiatrists, two nurses and one psychologist. The first author and third author were each trained and certified as a family psychoeducation instructor by the Japanese Network of Psychoeducation and Family Support Program, and they attended all sessions to insure the fidelity of the sessions. All staff except one nurse had participated in intensive training which consisted of more than six hours using the treatment manual of the Japanese Network of Psychoeducation and Family Support Program. The teaching materials for the relatives of the patients were two videotapes produced by the Department of Neuropsychiatry, Kochi Medical School [28], and a booklet developed by our department. At the first session, we gave the participants information on the causes and symptoms of major depression; at the second session, we provided information on drug treatment and psychosocial treatment; at the third session, we provided information on community resources; and at the fourth session, we provided guidelines for families caring for patients. After the lecture in each session, we provided supportive group therapy focusing on problem-solving skills for approximately 90 min. In the group therapy sessions, the participants were encouraged to give a narrative of their subjective experience in taking care of the MDD patient. Each session lasted approximately 2 hours. The groups met once every two weeks over the course of six weeks. The brief multifamily psychoeducation which we developed contained significant modifications from the McFarlane model. In the McFarlane model, clinically stable individuals with a psychiatric disorder participate in the family groups along with their caregiving relatives. We did not include patients with MDD in the family groups because the patients with MDD easily felt guilty. With regard to the structure of the multifamily group sessions, common steps in the problem-solving process shared by the standard model of the Japanese Network of Psychoeducation and Family Support Program and the McFarlane Model were: (i) socializing with other families, (ii) defining the problem or goal, (iii) listing all possible solutions suggested by the group members, and (iv) the family member who presented the problem chooses the solution that best fits the situation. However, the standard model of the Japanese Network of Psychoeducation and Family Support Program differs from the McFarlane Model with regard to several points. In the standard model of the Japanese Network of Psychoeducation and Family Support Program, the advantages and disadvantages of each solution are not discussed in detail, and an action plan to carry out the solution is not formed. The study took place between June 2008 and March 2010.

### Measures and Procedure

Self-administered questionnaires were used to measure the mental health state, care burden, Expressed Emotion and dysfunction of family life at the first and the last session of the family intervention.

#### K6

The K6 questionnaire is a six-item self-report questionnaire that was developed to screen for *DSM-IV *depressive and anxiety disorders within 30 days prior to its administration and which can also be used to quantify nonspecific psychological distress in general [29]. Each item is rated between 0 = "none of the time" to 4 = "all of the time", and the total score therefore ranges from 0 to 24. Two independent validation studies found the K6 to have an area under the receiver operating characteristic curve between 0.86 and 0.89 in predicting diagnoses of mental illness based on comprehensive diagnostic interviews [29,30]. The Japanese version of the K6 questionnaire also showed excellent efficacy in screening for anxiety and mood disorders in the Japanese general population, with an area under the receiver operating characteristic curve of 0.94 [31]. The optimal cut-off point of the K6 Japanese version has been proposed to be 9, with scores of 9 or higher indicating psychological distress [32]. Cronbach's α coefficient of reliability in the present study was 0.90.

#### The Japanese version of the Zarit Burden Interview short version (J-ZBI_8)

The Zarit Burden Interview is widely used to assess caregiver burden [33]. The Japanese version of the Zarit Burden Interview, J-ZBI, was developed by Arai, and the eight-item short version of the J-ZBI (J-ZBI_8) was also developed by Arai [34-36]. The items in the J-ZBI_8 are rated on a 5-point Likert scale (0 = never to 4 = very often) and the scores on the J-ZBI_8 range from 0 to 32. Cronbach's α of the J-ZBI_8 was 0.89, and the Pearson's correlation coefficient between scores on the J-ZBI and J-ZBI_8 was 0.93 [35]. Cronbach's α of J-ZBI_8 in the present study was 0.88.

#### Dysfunction in the Ordinary Family Life Scale (DFL)

The DFL was developed by Oshima, et al. [37] and reflects the dysfunction of the family life of caretakers. The DFL consists of 15 items, which are assessed on a 3-point Likert scale (0 = never to 2 = very often). The total score on the DFL ranges from 0 to 30. Cronbach's α was 0.89 among Japanese families of patients with schizophrenia [38], and 0.84 in the present study.

#### The Japanese version of the Family Attitude Scale (FAS)

The FAS, developed by Kavanagh, et al. [39], is a 30-item self-report inventory and measures families' Expressed Emotion (EE). Responses are totaled to give a score that ranges from 0 to 120, with higher scores indicating higher levels of burden or criticism [39]. A higher FAS rating significantly correlated with higher levels of criticism (r = 0.44), hostility (r = 0.41) and emotional overinvolvement (EOI), and with a higher level of emotional over-involvement (r = 0.27) in the Camberwell Family Interview (CFI) [40]. The Japanese version of the FAS, developed by Fujita et al. [41], showed excellent validity. The relative sensitivity and specificity of EE assessment with the FAS compared with the criticism component of the CFI were 100% and 88.5%, respectively [41]. Cronbach's α of FAS in the present study was 0.91.

### Ethical consideration

This study was approved by the Ethics Review Committee of Nagoya City University Graduate School of Medicine. All participants provided written informed consent after the purpose and procedures of the study were explained.

### Statistical analyses

Statistical analyses were performed using SPSS 17.0J software for Windows (SPSS, Chicago, IL, USA). Descriptive data analysis was conducted by calculating mean scores and standard deviation. We examined changes in scores on the K6, J-ZBI_8, DFL and FAS from baseline to the end of the intervention. Paired t-tests were used to determine changes in family scores, and a post-hoc sub-group comparison was performed using paired t-test with Bonferroni correction. The level of significance was set at p < 0.05. However, with regard to post-hoc analysis, the level of significance was set at p < 0.002, because we performed the paired t-tests twenty-four times. We calculated Cohen's d to quantify the effect size. The McNemar test was used to determine the significance of differences in the percentage of relatives who had a K6 score of over 9 points and relatives who had FAS score of over 60 points before and after the intervention.

## Results

### Characteristics of the participants

Of the 38 individuals who participated in the family psychoeducation groups during the study period, four were relatives of patients with bipolar disorder and were therefore not subjects of the present study. Two other individuals did not complete the questionnaires and were not included in the present study. The demographic characteristics of the 32 subjects are presented in Table 1. Among the 32 subjects, 27 subjects (84.3%) attended all four sessions, four (12.5%) attended three sessions, and one (3.1%) attended two sessions.

**Table 1:** Sample characteristics

		n	%
Gender			
	Male	9	28.1
	Female	23	71.9
family relationship			
	Fathers	2	6.2
	Mothers	12	37.5
	Hasbands	6	18.7
	Wives	8	25
	others	4	12.5
Parson living with a patient			
	Yes	22	68.7
	No	10	31.3
Inpatients or outpatients			
	Relatives of inpatients	14	43.8
	Relatives of outpatients	18	56.2
Age	(years)	61.7 ± 12.5	

### Mental Health Status

The K6 showed significant improvement from baseline to after the intervention (baseline = 8.6 ± 5.4, after intervention = 3.7 ± 3.3, paired-t = 5.9, P < 0.001, d = 1.1) (Table 2). The cut-off point in the K6 was 9 points. Sixteen relatives (50.0%) had a K6 score of over 9 points at baseline, while three relatives (9.3%) did so after the intervention (McNemar test, p < 0.0001).

**Table 2:** Differences between the baseline score and the after intervention score (paired t-test)

		Baseline		After intervention				
						
	n	M	SD	M	SD	T score	P	d
K6	32	8.6	5.4	3.7	3.3	5.9	< 0.001	1.1

J-ZBI_8	32	11.0	6.7	6.9	4.9	4.7	< 0.001	0.7

FAS	32	49.7	18.7	38.5	17.2	3.9	< 0.001	0.6

DFL	32	8.0	5.0	5.7	3.3	3.9	< 0.001	0.5

J-ZBI_8: The Japanese version of Zarit Burden Interview short version, FAS: Family Attitude Scale,

DFL: Dysfunction in the Ordinary Family Life Scale, d: effect size

### Care burden

The J-ZBI_8 showed significant improvement from baseline to after the intervention (baseline = 11.0 ± 6.7, after intervention = 6.9 ± 4.9, paired-t = 4.7, P < 0.001, d = 0.7). The DFL showed significant improvement from baseline to after the intervention (baseline = 8.0 ± 5.0, after intervention = 5.7 ± 3.3, paired-t = 3.9, P < 0.001, d = 0.5). The participants whose relatives were inpatients showed a significant improvement in the J-ZBI_8 score (baseline = 12.2 ± 6.1, after intervention = 6.8 ± 4.2, difference in mean scores = 5.4, paired-t = 4.7, P < 0.001), while the participants whose relatives were outpatients did not show a significant change in the J-ZBI_8 score (baseline = 10.1 ± 7.2, after intervention = 7.0 ± 5.5, difference in mean scores = 3.1, paired-t = 2.4, P = 0.023).

### Families' Expressed Emotion

The FAS showed significant improvement from baseline to after the intervention (baseline = 49.7 ± 18.7, after intervention = 38.5 ± 17.2, paired-t = 3.9, P < 0.001, d = 0.6). The cut-off point in the FAS was 60 points. Nine participants (28.1%) had an FAS score of over 60 points, while 5 (15.6%) did so after the intervention (McNemar test, p = 0.344).

Female relatives showed a significant improvement in the FAS score from baseline to after the intervention (baseline = 55.1 ± 17.1, after intervention = 40.8 ± 17.8, difference in mean scores = 14.3, paired-t = 4.5, P < 0.001). However, the male relatives did not show a significant change in the FAS score (baseline = 36.0 ± 16.0, after intervention = 32.7 ± 15.0, difference in mean scores = 3.3, paired-t = 0.6, P = 0.561).

The participants whose relatives were inpatients showed a significant improvement in the FAS score (baseline = 55.6 ± 13.3, after intervention = 41.7 ± 16.5, difference in mean scores = 13.9, paired-t = 4.6, P < 0.0001), while the participants whose relatives were outpatients did not show a significant change in the FAS score (baseline = 45.2 ± 21.3, after intervention = 36.0 ± 17.8, difference in mean scores = 9.2, paired-t = 2.0, P = 0.056).

## Discussion

In the present pilot study, we administered brief multifamily psychoeducation consisting of four sessions over six weeks to relatives of patients with depressive disorder. Families participating in this program reported significant improvements in their mental health, care burden and expressed emotion.

The K6 score on the degree of mental health fell significantly after the intervention and this suggests that the mental health of relatives significantly improved during the short intervention period of six weeks. K6 is the standard that was developed to screen for anxiety and mood disorder, and it was reported that if the K6 score is more than nine points, the probability of having an anxiety or a mood disorder is 50% [32]. Sixteen relatives (50%) had a K6 score of more than nine points before the intervention, but only three (9.3%) did so after the intervention. This suggests that providing brief multifamily psychoeducation for relatives of patients with depressive disorder helps reduce the probability of a mental illness in the relatives themselves.

Furthermore, the care burden of relatives living with the patient, evaluated by the J-ZBI_8 and DFL, significantly improved after the intervention. This suggests that brief multifamily psychoeducation alleviates family care burden and the difficulty of family life at least in the eyes of the relatives.

The reduction in the FAS score indicates that the EE of relatives decreased through brief multifamily psychoeducation. Studies on EE have primarily been conducted on the families of patients with schizophrenia. The family's EE is a good predictor of whether a patient relapses, and an association between a high EE level and high rate of recurrence has been demonstrated [42]. On the other hand, with regard to depressive disorder, there have been fewer studies on the relationship between the family's EE and the course of the illness. Although Hayhurst et al. [43] reported that there was no clear association between the EE of a spouse and recurrence of depression in the patient, three studies reported that high EE predicted bad consequence [44-46].

Because brief multifamily psychoeducation reduced the FAS score on the degree of EE, it is thought that it would have a good influence not only on their families but also on the patients. Fabbri et al. [22] reported that the relapse rate was lower in the group who received family intervention than in the control group. In a future study, it is worth investigating whether brief multifamily psychoeducation to relatives of patients improves depressive symptoms and the QOL of patients.

In this study, the multifamily psychoeducation remarkably reduced the FAS score in women, but not in men. The baseline score of the FAS among women was significantly higher than that among men (55.1 ± 17.1 for women, 36.0 ± 16.0 for men). This may be due to the difference in gender-related expectations between men and women in Japanese society. Wittmund et al. [9] reported that female spouses seem to have a burden-related increased risk of depression, independent of the partner's type of illness. The reason why only the women's FAS score remarkably decreased in this study cannot be definitively determined, but it may be because women respond better to increased social support obtained through telling her experience to other participants and getting advice from the other participants in family psychoeducation. For male relatives, we must further refine methods of psychoeducation.

The change in the mean FAS score of the participants whose relatives were inpatients was 13.9, and that of participants whose relatives were outpatients was 9.2. Moreover, the change in the mean J-ZBI_8 score of the participants whose relatives were inpatients was 5.4, and that of participants whose relatives were outpatients was 3.1. Although the FAS and J-ZBI_8 scores at baseline did not show significant differences between relatives of inpatients and relatives of outpatients (FAS, 55.6 ± 13.3 in relatives of inpatients, 45.2 ± 21.3 in relatives of outpatients; J-ZBI_8, 12.2 ± 6.1 in relatives of inpatients, 10.1 ± 7.2 in relatives of outpatients), the relatives' burdens were numerically heavier for inpatients than for outpatients and were more responsive to our interventions.

This study has a number of obvious limitations due to its preliminary nature. The sample size was small and there was no control group. In addition, the present study evaluated the relatives only after the intervention, and the long-term consequences of this brief multifamily psychoeducation were unclear. The duration and severity of MDD in the patients varied. However, the results of this study are noteworthy, because the mental health, care burden and EE of relatives of patients with depressive disorder significantly decreased after our short intervention. This suggests that brief multifamily psychoeducation is a useful intervention to reduce the psychosocial burden of relatives of patients with MDD. It may even ameliorate the course of the illness in the patients themselves.

## Conclusions

In the present study, the mental health, care burden and expressed emotion of families participating in this program improved significantly. This suggests that brief multifamily psychoeducation is a useful intervention to reduce the psychosocial burden of the relatives of patients with depressive disorder. Further evaluation of our brief family psychoeducation for relatives of patients with major depression is warranted.

## Competing interests

FK, HIT, MK, MES, YM, AN, HIRT, MAS, NS, YK, and YY have no conflict of interest to declare. TAF has received research funds and speaking fees from Astellas, Dai-Nippon Sumitomo, Eli Lilly, GlaxoSmithKline, Janssen, Meiji, Otsuka, Pfizer, Schering-Plough and Yoshitomi. He is on the research advisory board of Sekisui Chemicals and Takeda Science Foundation. He has received royalties from Igaku-Shoin, Seiwa-Shoten, Nihon Bunka Kagaku-sha and American Psychiatric Publications. The Japanese Ministry of Education, Science, and Technology and the Japanese Ministry of Health, Labor and Welfare have also funded his research.

## Authors' contributions

FK participated in designing the study, performed the statistical analysis, and wrote the paper. FK also performed family intervention as a therapist. HIT and YY coordinated the study. HIT, MK, MES, YM, AN, HIRT, MAS, NS and YK performed family intervention as therapists. TAF supervised the study and edited various drafts of the paper. All authors have read and approved the final manuscript.

## Pre-publication history

The pre-publication history for this paper can be accessed here:

http://www.biomedcentral.com/1471-244X/11/56/prepub
